# Ciliary neurotrophic factor (CNTF) plus soluble CNTF receptor α increases cyclooxygenase-2 expression, PGE_2 _release and interferon-γ-induced CD40 in murine microglia

**DOI:** 10.1186/1742-2094-6-7

**Published:** 2009-03-06

**Authors:** Hsiao-Wen Lin, Mohit Raja Jain, Hong Li, Steven W Levison

**Affiliations:** 1 Department of Neurology and Neurosciences, UMDNJ-New Jersey Medical School Cancer Center, Newark, NJ, USA; 2 Department of Biochemistry and Molecular Biology, UMDNJ-New Jersey Medical School Cancer Center, Newark, NJ, USA

## Abstract

**Background:**

Ciliary neurotrophic factor (CNTF) has been regarded as a potent trophic factor for motor neurons. However, recent studies have shown that CNTF exerts effects on glial cells as well as neurons. For instance, CNTF stimulates astrocytes to secrete FGF-2 and rat microglia to secrete glial cell line-derived neurotrophic factor (GDNF), which suggest that CNTF exerts effects on astrocytes and microglia to promote motor neuron survival indirectly. As CNTF is structurally related to IL-6, which can stimulate immune functions of microglia, we hypothesized that CNTF might exert similar effects.

**Methods:**

We performed 2-D and 1-D proteomic experiments with western blotting and flow cytometry to examine effects of CNTF on primary microglia derived from neonatal mouse brains.

**Results:**

We show that murine microglia express CNTF receptor α (CNTFRα), which can be induced by interferon-γ (IFNγ). Whereas IL-6 activated STAT-3 and ERK phosphorylation, CNTF did not activate these pathways, nor did CNTF increase p38 MAP kinase phosphorylation. Using 2-D western blot analysis, we demonstrate that CNTF induced the dephosphorylation of a set of proteins and phosphorylation of a different set. Two proteins that were phosphorylated upon CNTF treatment were the LYN substrate-1 and β-tubulin 5. CNTF weakly stimulated microglia, whereas a stronger response was obtained by adding exogenous soluble CNTFRα (sCNTFRα) as has been observed for IL-6. When used in combination, CNTF and sCNTFRα collaborated with IFNγ to increase microglial surface expression of CD40 and this effect was quite pronounced when the microglia were differentiated towards dendritic-like cells. CNTF/sCNTFRα complex, however, failed to increase MHC class II expression beyond that induced by IFNγ. The combination of CNTF and sCNTFRα, but not CNTF alone, enhanced microglial Cox-2 protein expression and PGE_2 _secretion (although CNTF was 30 times less potent than LPS). Surprisingly, Cox-2 production was enhanced 2-fold, rather than being inhibited, upon addition of a gp130 blocking antibody.

**Conclusion:**

Our studies indicate that CNTF can activate microglia and dendritic-like microglia similar to IL-6; however, unlike IL-6, CNTF does not stimulate the expected signaling pathways in microglia, nor does it appear to require gp130.

## Background

Microglia are the resident immune cells of the CNS and they exert innate and adaptive immune functions like peripheral macrophages. Normally microglia display a ramified morphology and they act as support cells. When nervous system homeostasis is disturbed by hazardous stimuli, like viruses, bacteria or traumatic injury, microglia become activated and are capable of secreting an array of soluble factors that include cytokines, chemokines and reactive nitrogen and oxygen species. Activated microglia can also act as phagocytes to engulf tissue debris and dead cells [1]. They may also become antigen presenting cells (APCs), which present antigenic peptides mounted on major histocompatibility complex (MHC) molecules to T lymphocytes to stimulate a cascade of T cell responses [2-4]. These immune properties of microglia are exquisitely regulated by cytokines secreted from T cells. The Th1 cytokine, IFNγ can stimulate microglia to increase phagocytosis and expression of MHC class II and CD40 molecules [5-7], whereas Th2 cytokines, like IL4 and IL-10, can counter-act the effect of IFNγ on microglia [8,9]. Interactions between T cells and microglia are important determinants for the extent of inflammation in the CNS.

Multiple sclerosis (MS) is a T cell-mediated demyelinating disease of the CNS and the expression of antigen presenting molecules on microglia has a pivotal role in the development of MS. Cell-cell interactions mediated by MHC and co-stimulatory molecules, including CD40, B7.1 and B7.2 molecules, expressed on the microglia and T cell receptors (TCR) and specific counter receptors for the co-stimulatory molecules located on the surface of T cells are essential for optimal T cell-APC adhesion and reciprocal activation [10,11]. Studies on experimental autoimmune encephalomyelitis (EAE), an animal model for MS, show that microglial activation precedes the onset of disease symptoms and the activated microglia exhibit increased expression of MHC class II, CD40 and B7 molecules [12].

In addition, activated microglia may also express cyclooxygenases (Cox), which are enzymes that generate prostanoids. Prostanoids, including prostaglandins and thromboxanes, are potent factors that can act on a variety of cells and have diverse actions [13]. However, these factors are short-lived and only act in a paracrine or autocrine manner. Cox-2 is the inducible form of Cox and it is rapidly expressed by microglia in response to injury. Whereas Cox-2 expression is undetectable in microglia in healthy subjects, there is a significant induction of Cox-2 in chronic active MS lesions [14]. Cox-2 expression has been identified in macrophages/microglia adjacent to damaged oligodendrocytes, suggesting that microglial expression of Cox-2 is involved in the development of demyelination. The metabolites of Cox, prostaglandin D (PGD) and PGE, are at higher concentrations in cerebrospinal fluid (CSF) of MS patients in active disease state compared to healthy controls [15,16]. Concentrations of PGE increase sharply before the onset of clinical symptoms and drop during deterioration to return to basal levels [17]. These studies suggest that the production of Cox-2 and PGE closely correlate with the development of MS.

Brain cells can also produce cytokines that modify the extent and nature of neuroinflammatory responses. Ciliary neurotrophic factor (CNTF), a member of the interleukin-6 family of cytokines, is produced following brain injury by astrocytes. Named on the basis of its initially characterized bioactivity, CNTF directly supports the survival of a variety of neuronal populations [18-24]. In addition, CNTF activates astrocytes, promoting their capacity to support neurons and oligodendroglia [25,26]. However, the effects of CNTF on microglia have been only partially studied [27-29]. Transgenic mice studies have shown that CNTF is required to maintain motor neurons after birth because CNTF knockout mice develop a progressive loss and atrophy of motor neurons and exhibit reduced muscle strength in adulthood although they are fully viable and developmentally normal [30]. Whereas CNTF is regarded as an important injury induced cytokine, cardiotrophin-like cytokine (CLC), which is a structurally related factor with CNTF and binds to CNTF receptor-α (CNTFRα) leading to activation of gp130, LIF receptor and STAT3 [31], is developmentally important. Similar to CNTFRα knockout mice, animals with CLC deletion die as neonates from loss of motor neurons affecting the facial nucleus [32].

In particular, CNTF has been shown to reduce the symptoms of EAE and the absence of CNTF exacerbates the severity of MS disability. MS patients with CNTF null mutations develop disease symptoms at earlier ages with more severe motor disabilities and more relapses compared to individuals who are CNTF heterozygotes [33]. Similar results are also seen in CNTF knockout mice induced with EAE [34]. Whereas individuals with a CNTF null mutation develop earlier and more severe disease, being a CNTF null or heterozygote is not a risk factor for developing MS. On the other hand, intravenous injections of CNTF induce acute-phase responses in rat liver cells with increased expression of β-fibrinogen, α-1-antichymotrypsin and α-2-macroglobulin [35,36]. These effects suggest that CNTF might regulate immune responses within the CNS as well.

Several studies have shown that injecting CNTF directly into the neocortex induces several features of gliosis where astrocytes become hypertrophic with increased GFAP expression and microglia become more ameboid [29,37,38]. Studies on cultured rat microglial cells have shown that CNTF can induce low-affinity nerve growth factor receptor and CD4 expression [27], suggesting that CNTF can exert direct effects on microglial cells. Indeed, we have recently reported that CNTF-treated rat microglia secreted soluble factors that increase motor neuron survival [39]. The goal of the current studies was to elucidate how CNTF regulates the immune functions of murine microglia.

CNTF belongs to the IL-6 cytokine family, which includes leukemia inhibitory factor (LIF), cardiotrophin-1 (CT-1), oncostatin-M (OSM) and IL-11. IL-6 family cytokines share glycoprotein 130 (gp130) as a common signal transducer, and similar to other IL-6 cytokines, CNTF may also signal through Janus Kinase (Jak)/signal transducers and activators of transcription (STAT) and the mitogen-activated protein kinase (MAPK) pathways. The canonical CNTF receptor is a tripartite complex composed of the binding protein for CNTF, the CNTF receptor α (CNTFRα), the LIF receptor (LIFR) and gp130 [40]. CNTFRα is linked to the cell membrane through a glycosyl phosphatidylinositol (GPI) linkage and like other GPI-linked receptors it can be cleaved by phosphatidylinositol-specific phospholipase C (PI-PLC) to create a freely soluble receptor [41]. The complex formed by sCNTFRα and CNTF can, therefore, serve as agonists for cells that do not express CNTFRα.

In this study, we first examined whether murine microglia express CNTFRα. Then, we stimulated microglia with CNTF alone or in combination with soluble CNTFRα to determine how the CNTFRα signals. We also investigated whether CNTF regulates antigen presenting molecules and prostaglandins produced by microglia. Cumulatively, our studies indicate that CNTF can activate microglia similar to IL-6, however, unlike IL-6, CNTF does not stimulate expected signal transduction pathways nor does CNTF appear to require gp130 to affect microglia.

## Methods

### Materials

Recombinant rat CNTF (rrCNTF) was purchased from Alomone (C-245, Jerusalem, Israel), and recombinant mouse IL-6 (rmIL-6, 406-ML-005), recombinant mouse IL-6 receptors (rmIL-6R, 1830-SR-025), recombinant mouse interferon-γ (rmIFNγ, 485-MI-100) and recombinant mouse granulocyte-macrophage colony-stimulating factor (rmGMCSF, 415-ML-010), were purchased from R&D Systems (Minneapolis, MN). Recombinant mouse leukemia inhibitory factor (rmLIF, LIF2010) was purchased from Chemicon (Temecula, CA). COX-2 antibody (160126, lot 155350-1) was purchased from Cayman Chemical (Ann Arbor, Michigan) and CNTFRα antibody (558891) was purchased from BD Pharmingen. β-Tubulin antibody (sc-9104) was purchased from Santa Cruz Biotechnology, Inc (Santa Cruz, CA). STAT3 (9132), phospho-STAT3-tyr705 (9131), phospho-ERK1/2 (9101) and ERK1/2 (9102) antibodies were purchased from Cell Signaling Technologies (Beverly, MA). Phospho-tyrosine/serine/threonine antibody was purchased from AbCam (ab15556, Cambridge, MA). Horseradish peroxidase (HRP)-conjugated secondary antibodies were purchased from Jackson ImmunoResearch Laboratories (West Grove, PA).

### Enriched microglial cultures

Primary mixed glial cultures were prepared from P0-2 mouse brains. Briefly, C57BL/6 mouse pups were sacrificed by decapitation and the whole brains excluding cerebellums and olfactory bulbs were isolated. The meninges were removed, tissues were enzymatically digested using Accutase (AT104, Innovative Cell Technologies, San Diego, CA) and mechanically dissociated, and the cell suspension was passed through 100 μm cell strainers and centrifuged at 1,500 rpm for 7.5 min. Cells were counted using a hemocytometer in the presence of 0.1% trypan blue and plated into 75 cm^2 ^tissue culture flasks at a density of 2 × 10^5 ^viable cells/cm^2 ^in minimum essential medium (MEM, 11090-99, Invitrogen, Carlsbad, CA) supplemented with 10% fetal bovine serum (FBS), 2 mM glutamine, 100 U/100 μg/ml penicillin/streptomycin and 0.6% glucose (MEM-10C). Medium was changed every 3 days after plating. On day 9, the mixed glial cultures were shaken on an orbital shaker at 250 rpm for 60–75 minutes to dislodge microglial cells. The nonadherent cells after shaking were plated onto 6-well or 12-well plates at 8 × 10^4 ^viable cells/cm^2^, and incubated in 37°C for 30 min to allow microglial cells to adhere. The wells were rinsed extensively with MEM to eliminate nonadherent cells and debris. The enriched microglial cultures were fed with 2 mL/well in 6 well plates or 1 mL/well in 12 well plates. The medium contained MEM supplemented with 1% FBS, 0.66 mg/ml BSA, 100 μg/ml d-biotin, 5 ng/ml insulin, 1 ng/ml selenium, 40 μg/ml iron poor transferrin, 2 mM glutamine, 15 mM HEPES buffer, and 100 U/100 μg/ml penicillin/streptomycin (MCDM). Enriched microglia were treated with cytokines ~18 hours after plating. Purity of the enriched murine microglial cultures was confirmed to be > 99% by CD11b and A2B5 staining.

### Protein isolation

After cytokine treatments, microglial cells were washed three times with PBS, then lysed in buffer containing a final concentration of 1% Triton-X-100, 10 mM Tris-HCl, pH 8.0, 150 mM NaCl, 0.5% nonidet P-40, 1 mM EDTA, 0.2% EGTA, 0.2% sodium orthovanadate and 1 μL/mL protease inhibitor cocktail (P8340-1 ML, Sigma, St. Louis, MO). Samples were rocked at 4°C for 15 minutes. DNA was sheared using a 21-gauge needle prior to centrifugation at 10,000 rpm for 10 min at 4°C. Protein concentrations from the supernatants were determined using the BCA colorimetric assay (Pierce, Rockford, IL). Protein lysates were aliquoted and stored at -20°C until needed.

### Enzyme-linked immunosorbent assay (ELISA)

Prostaglandin E_2 _(PGE_2_) content in the supernatant was assayed by ELISA developed with commercially available PGE_2 _ELISA kit purchased from Caymen Chemical (514010). Cells were stimulated with rrCNTF (5 ng/mL) and sCNTFRα (200 ng/mL) or left untreated for 18 hours. Supernatants were collected and frozen in -20°C until assayed.

### Western blotting

Ten to fifteen micrograms of protein isolated from the microglial cells was separated on 7% Tris-Acetate polyacrylamide gels (Invitrogen), electrophoresed at 150 V for 80 minutes, and transferred at 300 mA for 80 minutes to nitrocellulose membranes (LC2000, Invitrogen). Membranes were stained with 0.1% Ponceau S in 5% acetic acid to confirm proper transfer of proteins. Then, membranes were blocked for 1 hour in 10% milk diluted in 0.05% Tween-20 in PBS (PBS-T). Membranes were incubated overnight at 4°C in primary antibody diluted 1% BSA/PBS-T. Following incubation with the primary antibody, the blot was extensively washed with PBST for 30 minutes and then incubated for 1.5 hours at room temperature with secondary antibody conjugated to HRP diluted in 1% BSA/PBST. The membrane was then washed extensively in PBS-T for 30 minutes prior to visualization using Renaissance™ Chemiluminescence (NEL104, NEN Life Science, Boston, MA). For stripping antibodies off western blots, membranes were incubated in stripping buffer (62.5 mM Tris (pH 6.8), 2% SDS and 100 mM 2-mercaptoethanol) for 15 minutes in a water bath at 50°C with shaking. Membranes were washed with PBS-T for 10 minutes and then blocked in 10% milk/PBS-T. COX-2 antibody was diluted 1/200, antibodies from Cell Signaling were diluted in 1/1,000 and HRP-conjugated donkey rabbit secondary antibody was used at 1/10,000. Images were obtained and quantified using a UVP imaging system with LabWorks software (UVP, Upland, CA).

### Flow cytometry

The enriched murine microglia were treated with cytokines for 24 hours and cells were incubated in Accutase to detach cells followed by MEM-10C and scraped. Cells were washed twice with FACS buffer containing Ca2+, Mg+ free PBS, 0.5% BSA and 0.02% sodium azide. Fc antibody (553142, BD Biosciences, San Jose, CA) diluted 1/50 was used to block Fc receptors by addition for 10 minutes on ice. MHC class II antibody conjugated with FITC (553551, BD Biosciences) diluted 1/25 and CD40 antibody conjugated with APC (558695, BD Biosciences) diluted 1/100 were incubated with cells for 1 hour on ice in dark. Cells were washed twice and fixed in 1% paraformaldehye. Ten thousand cells were measured on BD FACSCalibur in the UMDNJ Flow Cytometry Core Facility (Newark, NJ) using the Cell Quest program. Samples were prepared in triplicate and geometric mean and percent positive cells were calculated after correcting for non-specific binding using cells stained with isotype control antibodies.

### 2D-PAGE analysis

After cytokine treatment, cells were thoroughly washed with 0.5 × PBS and then lysed by sonication in isoelectric focusing rehydration buffer (7 M urea, 2 M thiourea, 4% CHAPS, 100 mM DTT, 0.2% Biolytes (pH 5–8), 0.01% Bromophenol Blue and protease inhibitor). One hundred micrograms of protein in a total of 185 μL of rehydration buffer was applied to 11 cm Biorad ReadyStrip IPG Strips (pH 5–8) for overnight rehydration. First-dimension isoelectric focusing was carried out on a Biorad PROTEAN IEF System at the UMDNJ Center for Advanced Proteomics Research , as described by the manufacturer for a total focusing time of 75000 VH. The strips were equilibrated with a solution containing 6 M urea, 0.375 M Tris-HCl, pH 8.8, 2% SDS, 20% glycerol, 2% (w/v) DTT for 15 min. The strips were further equilibrated with a solution containing 6 M urea, 0.375 M Tris-HCl, pH 8.8, 2% SDS, 20% glycerol, 2.5% (w/v) iodoacetamide for 15 min and directly applied to a 12.5% isocratic SDS-PAGE gel for electrophoresis. The resulting gel was then fixed (10% Acetic Acid and 40% Ethanol) for 30 min and stained overnight with SYPRO Ruby. Gels were destained (10% Methanol, 7.5% Acetic Acid) for 60 min. After washing with water, gels were scanned on a 9400 Typhoon Variable Mode Imager (GE Healthcare, Inc., Piscataway, NJ) using a Green (532) Laser and 610BP30 emission filter. For phospho-specific immunoblotting, samples were run in parallel and after the second dimension; proteins were transferred to nitrocellulose membranes at 100 V for two hours. Phosphorylated proteins were detected using a Phosphoserine/threonine/tyrosine antibody using standard western blotting procedures.

### Statistical analysis

Data were analyzed using one-way ANOVA followed by Tukey's post-hoc test or Student's t-test to detect significant differences between the means with p < 0.05. Where the post-hoc test was used for multiple comparisons, superscript letters indicate significant differences; that is, means with the same letter are not significantly different. For example, a bar with the letter *a *is not statistically different from one with the letters *ab*, but is different from a bar with the letter *b*.

## Results

### Microglia express CNTF receptor α (CNTFRα), which can be induced by IFNγ

**Figure 1:**
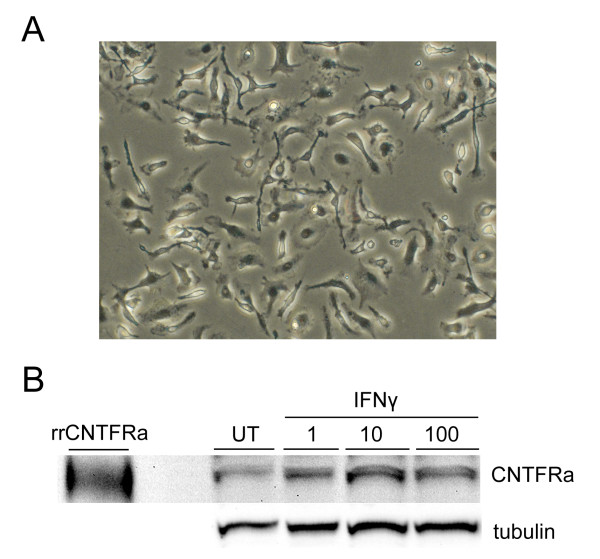
**Murine microglia express CNTFRα, which is induced further by IFNγ**. Panel **A **shows a representative photograph of cultured murine microglia. Panel **B **shows representative western blots for CNTFRα proteins in murine microglial cultures induced by IFNγ. Microglia were treated with IFNγ 1, 10 and 100 ng/mL, or left untouched for 22 hours. Forty micrograms of protein lysates were analyzed by western blotting. Membranes were probed with CNTFRα antibody and were re-probed with β-tubulin antibody to confirm equivalent protein loading. Recombinant rat soluble CNTFRα was loaded as a positive control (leftmost lane).

To investigate whether CNTF modulates microglial functions, we first asked whether murine microglia express receptors for CNTF. The CNTF receptor complex has been described as the CNTFRα, LIFR and gp130 and microglia are known to express LIFR and gp130 [42]. Therefore, we assessed expression of CNTFRα proteins. The pro-inflammatory cytokine, IFNγ is a strong stimulator for microglia and it primes microglia for antigen presentation by increasing expression of a number of cell surface receptors [2,43]. Therefore, we tested the hypothesis that IFNγ would upregulate CNTFRα expression in microglia. Primary microglial cultures were prepared and confirmed to be greater than 99% homogenous for CD11b. Unstimulated microglia or microglia that were stimulated with murine IFNγ at 1, 10 and 100 ng/mL for 22 hours were analyzed. Forty micrograms of total protein lysate were separated by gel electrophoresis, transblotted to nitrocellulose membranes, probed with CNTFRα antibody and then probed with β-tubulin antibody to confirm equivalent loading of proteins. These studies revealed that murine microglia expressed a protein that has a molecular weight of approximately 50 kDa (similar to that of recombinant rat CNTFRα) that reacts with antibodies raised against recombinant murine CNTFRα (Fig 1). Interestingly, the IFNγ dose response was bell-shaped, with IFNγ increasing the levels of CNTFRα protein with a maximum effect seen with 10 ng/mL of IFNγ.

### CNTF does not activate STAT and ERK pathways in murine microglia

**Figure 2:**
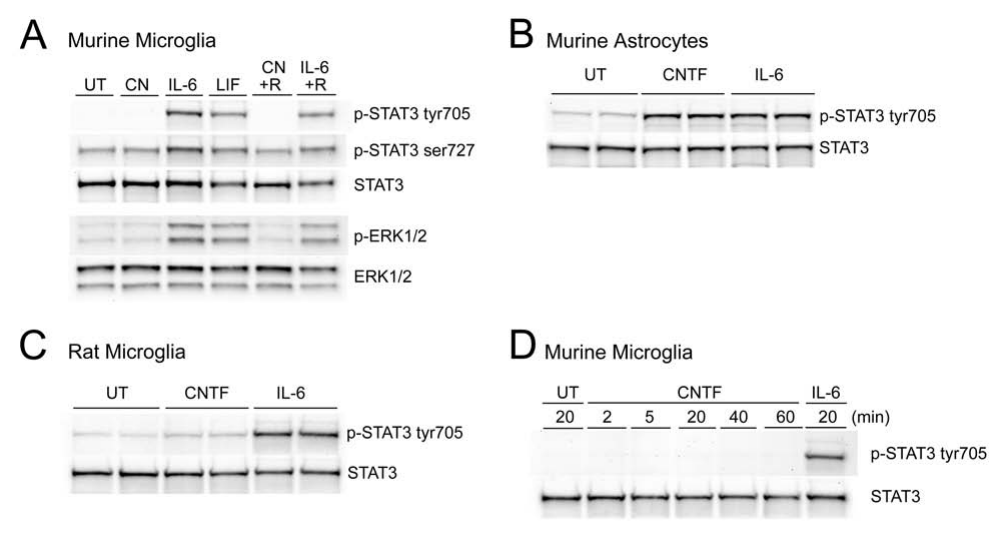
**CNTF does not activate STAT or ERK pathways in murine microglia**. After cytokine treatment, ten micrograms of protein lysates were analyzed by western blotting for phosphorylated proteins and the membranes were striped and reprobed with antibody against total protein. **A**, Enriched murine microglial cultures were treated with CNTF (10 ng/mL), IL-6 (5 ng/mL), LIF (10 ng/mL), CNTF (10 ng/mL) plus soluble CNTFRα (200 ng/mL), IL-6 (5 ng/mL) plus soluble IL-6R (200 ng/mL), or left untouched (UT) for 20 minutes. **B**, Murine astrocytes were treated with CNTF (10 ng/mL) or IL-6 (5 ng/mL) or left untreated for 20 minutes. **C**, Enriched rat microglial cultures (purity > 99%) were treated with CNTF (10 ng/mL) or IL-6 (10 ng/mL) or left untreated for 20 minutes. **D**, Murine microglia were treated with CNTF (10 ng/mL) for 2, 5, 20, 40 and 60 minutes or IL-6 (5 ng/mL) or left untreated for 20 minutes. Data are representative of 3 independent experiments.

Since murine microglia express the CNTFRα and our previous studies showed that murine microglia expressed gp130 as well as the LIF receptor-β (Lin *et al*., 2008, in revision for Journal of Neurochemistry), and as CNTF activates JAK/STAT and Ras/Raf/MAPK pathways in neurons and astrocytes [44,45], we asked whether STAT3 and ERK would be activated by CNTF. Enriched murine microglial cultures were stimulated with CNTF, CNTF plus sCNTFRα, IL-6, IL-6 plus sIL-6R or LIF, or left untreated for twenty minutes (Fig 2A). Ten micrograms of total protein were analyzed by western blotting for levels of phosphorylated STAT3 and ERK. IL-6 alone, IL-6 plus sIL-6R and LIF increased the phosphorylation of STAT3, most strongly at tyrosine 705 (tyr705) residue and to a milder degree at ser727 residue. ERK proteins were also phosphorylated in response to IL-6, IL-6 plus sIL-6R and LIF stimulation. In distinct contrast, neither CNTF nor the combination of CNTF and sCNTFRα increased phosphorylation of STAT3 or ERK.

Since recombinant murine CNTF is not commercially available, recombinant rat CNTF (rrCNTF) was used in our experiments. To confirm that rrCNTF binds to murine CNTFRα to activate JAK/STAT pathways, enriched murine astrocyte cultures were stimulated with rrCNTF and rmIL-6 for twenty minutes and STAT3 phosphorylation was assessed. Both rrCNTF and rmIL-6 increased phosphorylation of STAT3 in murine astrocytes (Fig 2B). To confirm that CNTF does not activate STAT3 in microglia, we also stimulated enriched rat microglial cultures with rrCNTF and rrIL-6 for twenty minutes and examined STAT3 phosphorylation. Again, rrCNTF failed to induce STAT3 phosphorylation in rat microglia while IL-6 stimulated strong phosphorylation of STAT3 tyr705 (Fig 2C). To determine whether the failure of CNTF to phosphorylate STAT3 was due to a slower recruitment of the receptors, we stimulated murine microglia with rrCNTF for 2, 5, 20, 40 and 60 minutes or rmIL-6 for 20 minutes. rrCNTF did not increase STAT3 phosphorylation at any time point examined whereas rmIL-6 stimulation strongly increased STAT3 phosphorylation compared to untreated cells (Fig 2D).

### CNTF treatment results in protein phosphorylation and dephosphorylation in murine microglia

**Figure 3:**
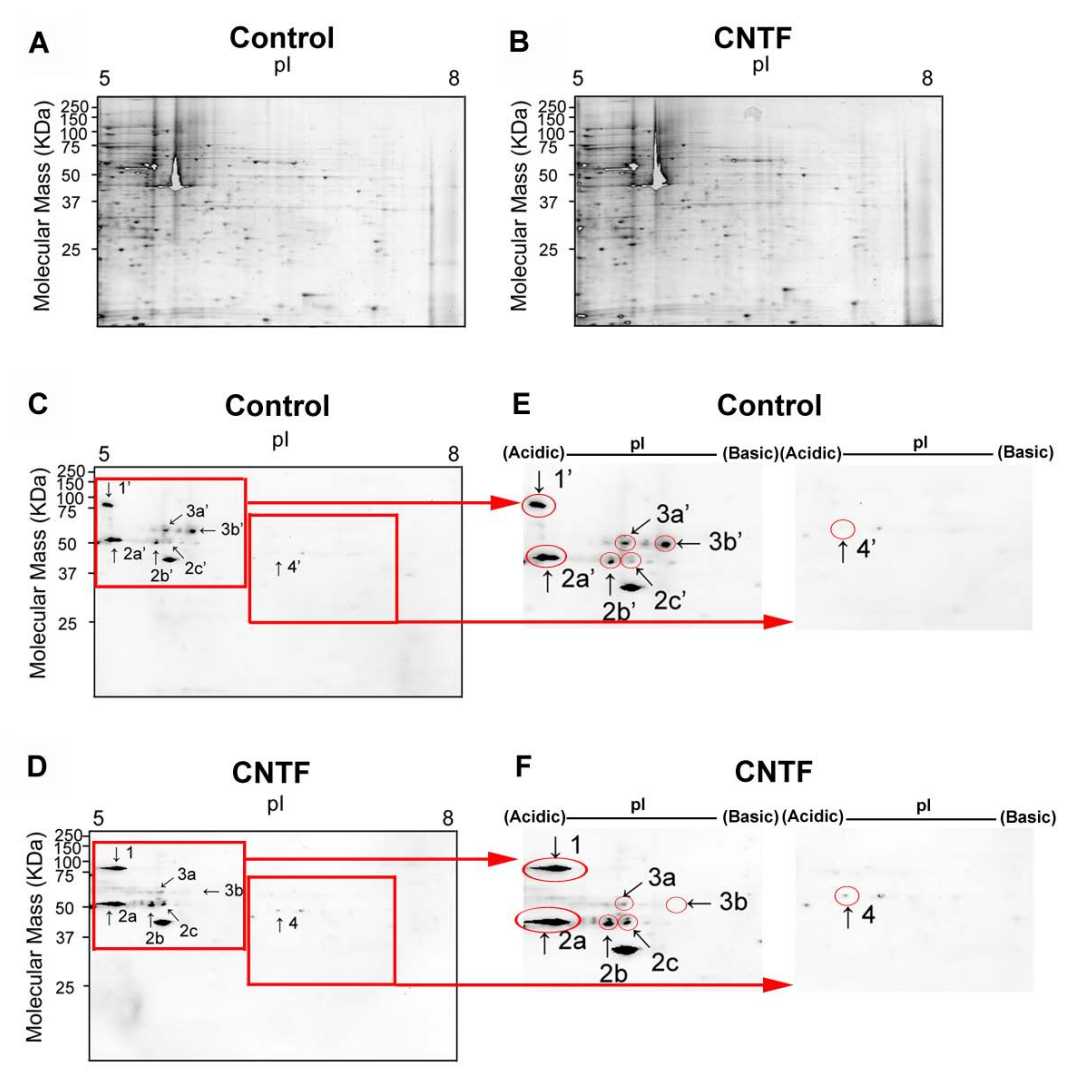
**CNTF treatment results in protein phosphorylation and dephosphorylation in murine microglia**. Enriched murine microglia were treated with CNTF (10 ng/mL) for 20 minutes or left untreated. One hundred micrograms of protein lysate were separated by isoelectrofocusing and subsequent SDS/PAGE under reducing conditions. Gels were fixed, stained with SYPRO Ruby, destained and then scanned on a 9410 Typhoon Imager. Panel **A **shows SYPRO Ruby staining of untreated microglia, and Panel **B **shows SYPRO Ruby staining of CNTF-stimulated microglia. For phosphospecific-immunoblotting, duplicate gels were transferred to nitrocellulose membranes and probed with an antibody against phospho-tyrsoine/serine/threonine to detect phosphorylated proteins in both untreated **(C) **and CNTF-stimulated microglia **(D)**. Spots which showed altered phosphorylation are depicted at higher power for untreated **(E) **and CNTF stimulated microglia **(F)**. CNTF altered phosphorylation of several proteins. Mass spectral analyses identified that Spot 1 is the hemopoietic cell specific Lyn substrate-1 (gi:13938627) and Spots 2a, 2b and 2c are β-tubulin 5 (gi:7106439). Spots 3 and 4 were too low in quantity to be unidentified. Data are representative of 3 independent experiments.

To confirm that CNTF is altering intracellular signaling pathways in murine microglia, despite the fact that we did not see increased phosphorylation of STAT-3 or ERK, we performed 2D gel electrophoresis followed by western blot analysis for tyrosine/serine/threonine phosphorylation. Murine microglia were stimulated with CNTF (10 ng/mL) for 20 minutes or left untreated. One hundred micrograms of protein lysates were separated by electrophoresis on each gel and duplicated gels were generated. One gel from each condition was stained with SYPRO Ruby or used for phospho-protein analysis where proteins were transferred to nitrocellulose membranes. SYPRO Ruby staining revealed hundreds of proteins of varying molecular weights and isoelectric points (pI) (Fig 3A, B). Western blot analyses showed that CNTF altered the phosphorylation of several proteins. For instance, the intensity of spot 1 was greater in CNTF treated cells (Fig 3D, F), corresponding to spot 1' (Fig 3C, E) in untreated cells, which indicated increased phosphorylation subsequent to CNTF stimulation. Spots 2a, 2b and 2c (Fig 3D, F), corresponding to Spot 2a', 2b' and 2c' (Fig 3C, E), respectively, also appeared more abundant in CNTF treated samples, indicating increased phosphorylation. Spot 3a and 3b (Fig 3D, F), corresponding to spots 3a' and 3b' (Fig 3C, E), showed a dramatic decrease in signal, indicating that phosphorylation of the protein was greatly reduced by CNTF treatment (Fig 3C–F). Finally, CNTF increased phosphorylation of Spot 4 (Fig 3D, F), corresponding to Spot 4' in the control cells (Fig 3C, E). Mass spectral analyses established that Spot 1 is the hemopoietic cell specific Lyn substrate-1 (gi:13938627) and Spots 2a, 2b and 2c are forms of β-tubulin 5 (gi:7106439). Spots 3 and 4 were too low in quantity to be identified.

### CNTF in combination with sCNTFRα induces cyclooxygenase-2 (Cox-2) and prostaglandin E2 (PGE_2_) in microglia that is gp130-independent

**Figure 4:**
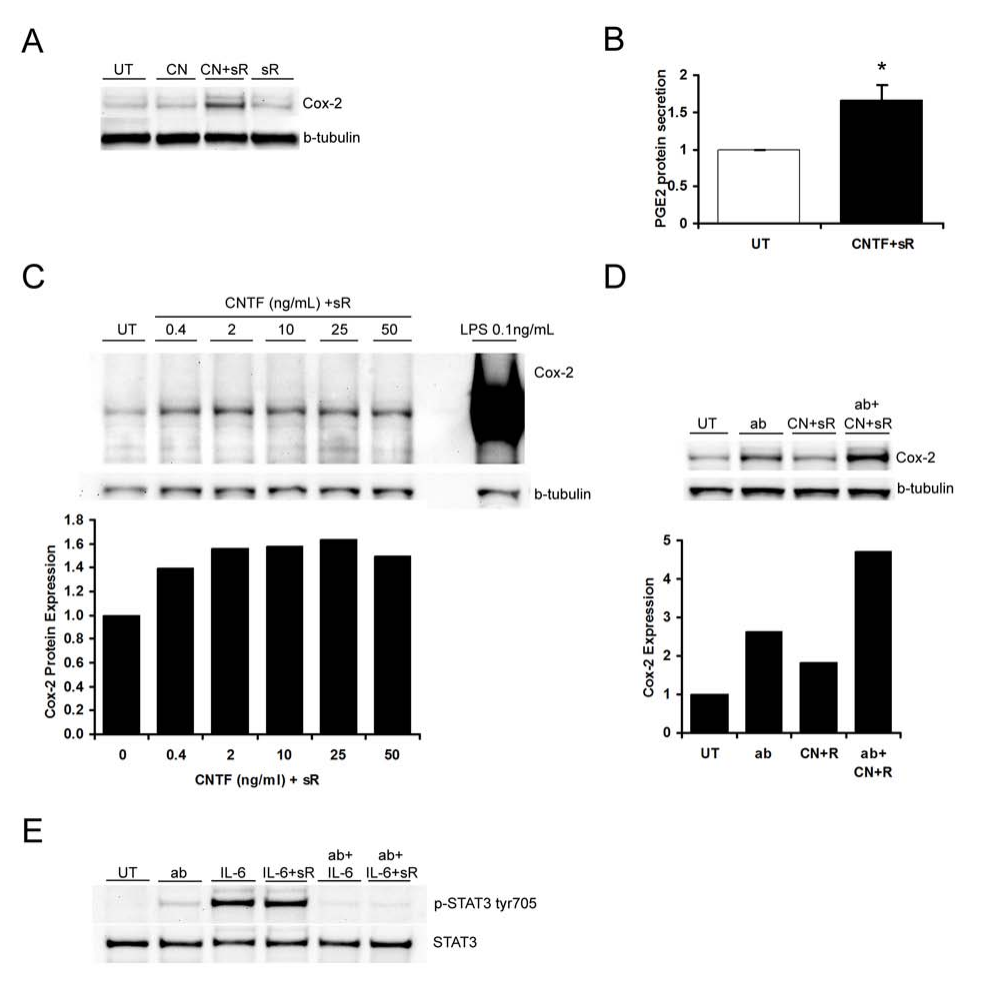
**The combination of CNTF and soluble CNTFRα increases Cox-2 and PGE_2 _production in murine microglia that is not inhibited by a gp130 function blocking antibody**. After cytokine treatment, cultured murine microglia were collected and 10 micrograms of protein lysates were analyzed by western blotting. **A**, Murine microglia cultures were treated with CNTF (10 ng/mL) (CN), the combination of CNTF (10 ng/mL) and soluble CNTFRα (200 ng/mL) (CN+sR), soluble CNTFRα (200 ng/mL) (sR), or left untouched (UT) for 16–18 hours. Membranes were probed with Cox-2 antibody and reprobed with β-tubulin antibody to confirm equivalent protein loading. Data are representative of 3 independent experiments. **B**, Murine microglia were treated with the combination of CNTF (5 ng/mL) and soluble CNTFRα (200 ng/mL) (CN+sR) or left untouched (UT) for 16–18 h. Supernatants were collected and analyzed by PGE_2 _ELISA. Values represent the means ± S.E.M. from 4 independent experiments. *p < 0.05 by Student's *t*-test. **C**, Murine microglia were treated with CNTF 0.4, 2, 10, 25 and 50 ng/mL in combination with soluble CNTFRα (200 ng/mL), or with LPS 0.1 ng/mL for 16–18 hours. Membranes were probed with Cox-2 antibody and reprobed with β-tubulin antibody. Data are representative of two independent experiments. **D**, Microglia were treated with gp130 antibody (5 μg/mL) (ab), the combination of CNTF (10 ng/mL) and soluble CNTFRα (200 ng/mL) (CN+sR), gp130 antibody for 1 hour followed by the combination of CNTF (10 ng/mL) and soluble CNTFRα (200 ng/mL) (ab+CN+sR), or left untreated (UT) for 18 h. Membranes were probed with Cox-2 antibody and reprobed with β-tubulin antibody. Data are representative of 4 independent experiments. **E**, Microglia were treated with gp130 antibody (5 μg/mL) (ab), IL-6 (5 ng/mL), a combination of IL-6 (5 ng/mL) and soluble IL-6R (200 ng/mL) (IL-6+sR), gp130 antibody for 1 hour followed by IL-6 (5 ng/mL) (ab+IL-6) or a combination of IL-6 (5 ng/mL) and soluble IL-6R (200 ng/mL) (ab+IL-6+sR), or left untreated (UT) for 20 minutes. Membranes were probed with phospho-STAT3 tyr705 antibody, stripped, and re-probed with STAT3 antibody.

Cox-2 is an inducible cyclooxygenase and is rapidly expressed by microglia in response to a variety of stimuli, and PGE_2 _is one of the metabolites produced by Cox-2. Therefore, the production of Cox-2 and PGE_2 _were examined after stimulating microglia with CNTF alone or in combination with sCNTFRα. Murine microglia were treated with CNTF (10 ng/mL), the combination of CNTF (10 ng/mL) and sCNTFRα (200 ng/mL) or sCNTFRα (200 ng/mL), or left untreated for 16–18 hours. Ten micrograms of total protein were analyzed by western blotting for Cox-2 expression. The murine microglia in these studies had low basal expression of Cox-2 as would be expected. The combination of CNTF and sCNTFRα increased Cox-2 expression approximately 2 fold. By contrast, neither CNTFR nor sCNTFRα alone had any effect (Fig 4A). After microglia were treated with CNTF and sCNTFRα for 16 hours, the supernatants were collected and analyzed for PGE_2 _content. PGE_2 _secretion was increased 1.7 fold (p < 0.05) by the combination of CNTF and sCNTFRα compared to untreated cells (Fig 4B). Microglia were stimulated with 0, 0.4, 2, 10, 25 or 50 ng/mL of CNTF with sCNTFRα (200 ng/mL), or stimulated with LPS at 0.1 ng/mL for 16–18 hours, and Cox-2 protein expression was analyzed by western blotting. Microglial production of Cox-2 in response to CNTF was dose dependent with the maximum effect seen at 2 ng/mL of CNTF. Thus, the ED50 for this induction is around 0.2 ng/mL (Fig 4C). Compared to LPS CNTF/sCNTFRα is a weak inducer of Cox-2 in that CNTF at 2 ng/mL plus sCNTFRα at 200 ng/mL increased Cox-2 by 1.6 fold while LPS at 0.1 ng/mL increased Cox-2 by 54 fold.

CNTF is a member of the IL-6 family of cytokines, which share gp130 as a common signaling molecule. To determine whether Cox-2 induction by CNTF/sCNTFRα requires gp130, we treated microglia with neutralizing antibodies against murine gp130 (5 μg/mL, about 10 times of ND50 based on manufacturer's information), the combination of CNTF (10 ng/mL) and sCNTFRα (200 ng/mL), or gp130 antibody (5 μg/mL) for 1 h followed by stimulation of CNTF (10 ng/mL)/sCNTFRα (200 ng/mL) for 16–18 hrs. In these experiments, neutralizing gp130 activity promoted Cox-2 expression by 2.5 fold compared to unstimulated cells (Fig 4D). CNTF/sCNTFRα induced Cox-2 by two fold compared to untreated cells. Unexpectedly, administering CNTF/sCNTFRα with gp130 antibodies further induced Cox-2 by two fold compared to stimulating with gp130 antibody alone. To confirm that the antibody blocks gp130 activity, we stimulated microglia with gp130 antibody (5 μg/mL), IL-6 (5 ng/mL), a combination of IL-6 (5 ng/mL) and soluble IL-6R (200 ng/mL), gp130 antibody for 1 hour followed by IL-6 (5 ng/mL) or a combination of IL-6 (5 ng/mL) and soluble IL-6R (200 ng/mL), or left cells untreated (UT) for 20 minutes. Western blot analysis of STAT3 phosphorylation showed that the gp130 antibody completely blocked IL-6-induced STAT3 phosphorylation as expected (Fig 4E).

### CNTF in combination with sCNTFRα potentiates the effect of IFNγ on CD40, but not MHC class II, expression in murine microglia

**Figure 5:**
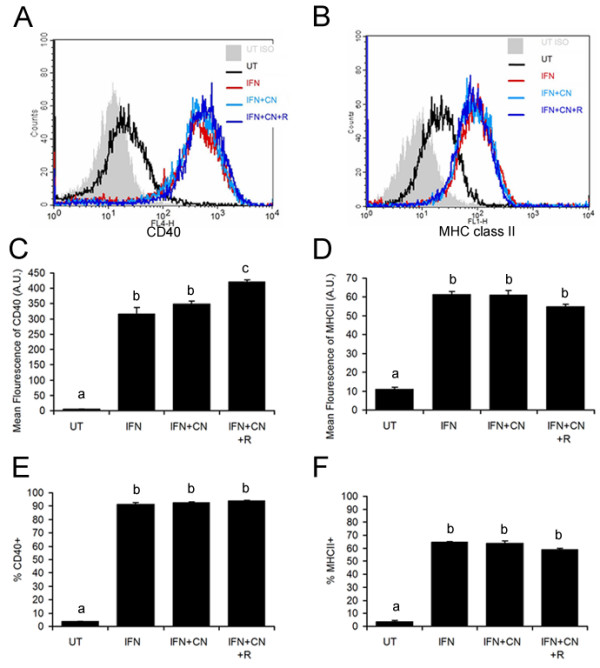
**CNTF in combination with sCNTFRα potentiates the effect of IFNγ on CD40, but not MHC class II, expression in murine microglia**. Microglia were treated with IFNγ (10 ng/mL) (IFN), IFNγ (10 ng/mL) plus CNTF (10 ng/mL) (IFN+CN), or IFNγ (10 ng/mL) plus the combination of CNTF (10 ng/mL) and sCNTFRα (200 ng/mL) (IFN+CN+R), or left untreated (UT) for 24 hours. 10,000 cells were analyzed by flow cytometry for CD40 and MHC class II expression. Panel **A **shows representative histograms for CD40 (APC) while Panel **B **shows representative histograms for MHC class II (FITC). **C**, Mean fluorescence of CD40 and inset shows averaged values from three independent experiments as normalized to IFNγ alone. **D**, Mean fluorescence of MHC class II; **E**, Percentage of CD40 positive cells; **F**, Percentage of MHC class II positive cells. Values represent the means ± S.E.M. from triplicates in one experiment. Different superscript letters indicate significant differences at the p < 0.05 levels as analyzed by one-way ANOVA followed by Tukey's post-hoc test. Data are representative of 3 independent experiments.

Antigen presenting and co-stimulatory molecule expression is required for microglia to induce T cell responses. Therefore, we tested the hypothesis that CNTF would regulate microglial expression of MHC class II and CD40. Enriched murine microglial cultures were stimulated with IFNγ alone, IFNγ plus CNTF or IFNγ plus the combination of CNTF and sCNTFRα, or cells were left untreated for twenty-four hours. Flow cytometry analysis showed that IFNγ strongly upregulated surface expression of CD40 on microglia and more than 90% of the cells were positive for CD40 (Fig 5). Stimulating with IFN plus CNTF showed a trend of increased CD40 expression compared to IFNγ alone, and an increase of approximately 10% in the mean fluorescence intensity (MFI). Interestingly, the combination of CNTF and sCNTFRα collaborated with IFNγ to increase CD40 expression by approximately 30% compared to IFNγ alone. On the other hand, IFNγ treatment increased MHC class II levels on microglia and approximately 60% of the cells were positive for MHC class II. However, combinatorial treatment with IFNγ and CNTF had no effect on MHC class II levels, neither MFI nor %MHC class II+, and adding sCNTFRα was no more effective.

### CNTF in the presence of sCNTFRα potentiates the effect of IFNγ on CD40, but not MHC class II, expression in dendritic-like microglia

**Figure 6:**
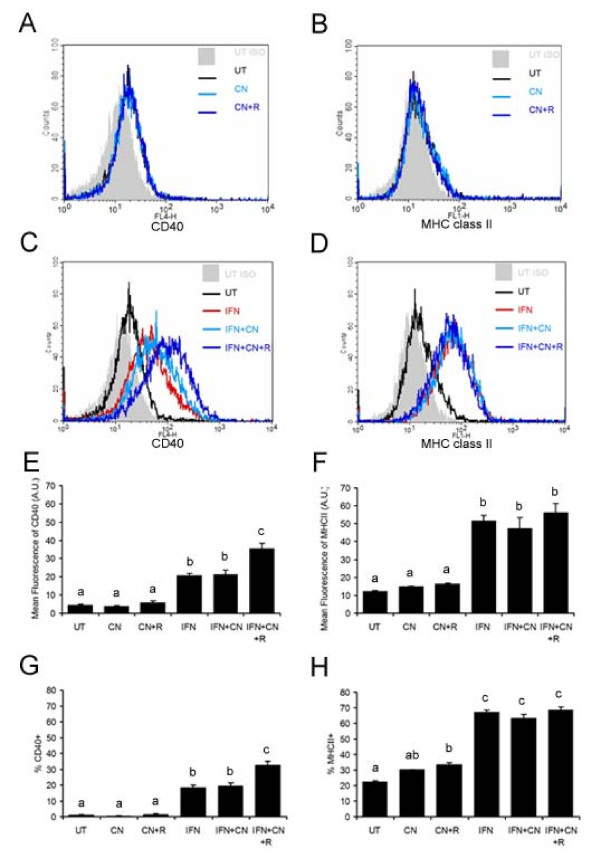
**CNTF in the presence of sCNTFRα potentiates the effect of IFNγ on CD40, but not MHC class II, expression in dendritic-like microglia**. After continuous growth in GMCSF (10 ng/mL) for 7 days, the dendritic-like microglia were treated with CNTF (10 ng/mL) (CN), a combination of CNTF (10 ng/mL) and sCNTFRα (200 ng/mL) (CN+R), IFNγ (10 ng/mL) (IFN), IFNγ (10 ng/mL) plus CNTF (10 ng/mL) (IFN+CN) or IFNγ (10 ng/mL) plus the combination of CNTF (10 ng/mL) and sCNTFRα (200 ng/mL) (IFN+CN+R) or left untreated (UT) for 24 hours. 10,000 cells were analyzed by flow cytometry for CD40 and MHC class II expression. Panel **A **and **C **show representative histograms for CD40 expression while Panel **B **and **D **show representative histograms for MHC class II expression. **E**, Mean fluorescence of CD40; inset shows data from three independent experiments, IFNγ, IFNγ plus the combination of CNTF and sCNTFRα. **F**, Mean fluorescence of MHC class II; **G**, Percentage of CD40 positive cells and inset shows data from three independent experiments. **H**, Percentage of MHC class II positive cells. Values represent the means ± S.E.M. from triplicates in one experiment. Different superscript letters indicate significant differences at the p < 0.05 levels as analyzed by one-way ANOVA followed by Tukey's post-hoc test. Data are representative of 3 independent experiments.

Granulocyte-macrophage colony stimulating factor (GMCSF) transforms microglia into dendritic-like cells [46]. Using CD11c as a dendritic cell marker, we established that stimulating murine microglia with GMCSF (10 ng/mL) for eight days resulted in 80% of these cells expressing CD11c. Using this culture paradigm, we tested the hypothesis that the combination of CNTF and sCNTFRα would also induce CD40 expression in dendritic-like cells. Microglia were treated with GMCSF for seven days and then stimulated with CNTF alone, CNTF plus sCNTFRα, IFNγ, IFNγ plus CNTF or IFNγ plus CNTF and sCNTFRα, or left untreated for twenty-four hours. By flow cytometry analyses, IFNγ increased CD40 expression in these dendritic-like cells, and the combination of CNTF and sCNTFRα, but not CNTF alone, collaborated with IFNγ to double CD40 expression as compared to IFNγ alone as measured by MFI and %CD40+ (Fig 6). IFNγ induced MHC class II in these cells, but neither CNTF nor the combination of CNTF and sCNTFRα further increased class II expression. In the absence of IFNγ, neither CNTF alone nor the combination of CNTF with sCNTFRα altered the levels of CD40 or MHC class II compared to untreated cells.

## Discussion

Studies on immunoreactivity for CNTFRα have shown that CNTFRα is most highly expressed by neurons and expressed by a subset of astrocytes [47-49]. Consistent with previous studies on rat microglia [39], here we show that murine microglia also express CNTFRα. The analyses reported herein further reveal that 1) the combination of CNTF and sCNTFRα, but neither alone, induces Cox-2 expression and PGE_2 _secretion from microglia; 2) Neutralizing antibodies against gp130 fail to inhibit CNTF/sCNTFRα-induced Cox-2, and neither CNTF nor CNTF/sCNTFRα activates canonical IL-6 signal transducers, including STAT3 and ERK; 3) CNTF increases the phosphorylation of the Lyn substrate-1 and β-tubulin 5; 4) The combination of CNTF and sCNTFRα collaborate with IFNγ to promote CD40, but not MHC class II, expression in microglia and especially in dendritic-like cells. Cumulatively, these data suggest that CNTF in combination with sCNTFRα serves as a weak pro-inflammatory signal to enhance the production of Cox-2, PGE_2 _and CD40 in microglia.

Our studies on murine microglia show that in the presence of soluble CNTFRα, CNTF increases Cox-2 production in a dose-dependent manner. Secretion of PGE_2_, as one of the metabolites produced by Cox-2 from microglia is also increased following CNTF/sCNTFRα stimulation. By contrast, Shapiro *et al*. showed that in human blood mononuclear cells, the combination of CNTF at 3 μg/mL and sCNTFRα suppressed PGE_2 _production and IL-6 showed similar inhibitory effects in their studies [50]. One explanation for this difference in response is that microglia are not simply macrophages that are residing within the CNS. Additionally, since human CNTF, at high concentrations (beyond 50 ng/mL), can bind to IL-6R and induces STAT3 activation [51] the inhibitory effect seen in the human mononuclear cells with CNTF may be a result of activating IL-6R. CNTF at nanogram doses does not activate STAT3, which suggests that it does not bind to IL-6R. Therefore, our studies indicate that in murine microglia, CNTF in combination with sCNTFRα promotes Cox-2 and PGE_2 _production.

The majority of studies to date indicate that CNTF binds to its specific CNTFRα to recruit LIFR and gp130, which leads to activation of JAK/STAT and Ras/Raf/MAPK pathways [40,44,45]. There is evidence that LIFR and IL-6R can also serve as α-receptors for CNTF [51-53]. Intriguingly, our studies on microglia strongly suggest that CNTF conveys its signal independent of gp130. It has been shown that p38MAPK mediates the LPS induced Cox-2 expression in microglia [54,55]. However, we did not observe evident phosphorylation of p38MAPK after stimulating murine microglia with CNTF (10 ng/mL)/sCNTFRα (200 ng/mL) for 20 minutes, while LPS (10 ng/mL) induced strong phosphorylation of p38MAPK (data not shown). Thus, it is unlikely, that our results are due to endotoxin contamination of the recombinant CNTF we utilized. Nevertheless, LPS is a much stronger inducer of Cox-2 than CNTF/sCNTFRα and thus, it may be that the CNTF/sCNTFRα induced p38MAPK phosphorylation is too low to be detected. Alternatively, it is possible that CNTF/sCNTFRα acts indirectly to induce Cox-2 via phospholipase A2 [56].

CNTF has previously been shown to exert pro-inflammatory actions. For instance, intravenous injections of CNTF induce acute-phase responses with increased expression of β-fibrinogen, α-1-antichymotrypsin and α-2-macroglobulin in rat liver cells [35,36]. CNTFRα is GPI-linked to cell membranes and is released from skeletal muscles after nerve injury, and the concentration of sCNTFRα is elevated in the CSF of patients with lupus, ALS and epilepsy [57,58]. These data suggest that sCNTFRα is an injury-induced signal and is involved in central and peripheral responses to injury. It has been shown that sCNTFRα alone or in combination with CNTF can serve as a chemoattractant for macrophages [59]. Thus, our results were not completely unexpected.

Although the conventional view of sCNTFRα is that it conveys signals of CNTF for cells that do not express CNTFRα [58], a recent study revealed that a complex formed between CNTF and sCNTFRα promotes neurite outgrowth and regulates expression of several neurotrophic genes on neurons, whereas CNTF alone was without effect [60]. In agreement with this study, our studies show that although microglia respond to CNTF, the complex formed by CNTF and sCNTFRα effectively promotes Cox-2, PGE_2 _and CD40 production in microglia whereas CNTF alone is with little effect. Interestingly, the effect of CNTF and sCNTFRα was more pronounced in the dendritic-like microglia than in the microglial cultures, which suggests that dendritic-like microglia respond more strongly to CNTF/sCNTFRα. Whereas, exogenous CNTF administration has been shown to exert protective effects in MS and EAE, our data and those of others would caution against the use of CNTF as a neuroprotective agent in that the inflammatory side effects subsequent to delivery may limit the clinical usefulness of administering CNTF in treating neurodegenerative diseases, especially where there is an inflammatory component.

Our data show that CNTF does not activate STAT3 and ERK pathways in microglia. In contrast, Krady *et al*. (2008) showed that rrCNTF elicits a modest increase in STAT3 phosphorylation in rat microglia [39]. Initially we surmised that the difference in responsiveness was species related, however, subsequent to careful analyses the discrepancy between those data and the data reported herein can be attributed to differences in the purity of the microglial cultures. Rat microglial cultures are typically enriched by incubating non-adherent cells obtained from mixed glial cultures on bacteriological dishes. Incubating rat microglia on bacteriological dishes for 10 minutes (our protocol) instead of 40 minutes (previous protocol) increases the purity of the microglial cultures from 90% to 99% as determined by CD11b and A2B5 staining. In these highly enriched rat microglial cultures STAT3 is not phosphorylated following CNTF treatment whereas in the less enriched microglial cultures CNTF induces STAT3 phosphorylation. This STAT3 signaling likely results from activation with the contaminating oligodendrocyte progenitors (OPCs) and, to a smaller degree, astrocytes. We also assessed whether soluble factors secreted from OPCs and/or astrocytes promote microglial STAT3 phosphorylation in response to CNTF. To do so, highly enriched rat microglia were cultured in oligodendrocyte or astrocyte-conditioned media overnight followed by CNTF treatment for 20 minutes. As expected, CNTF failed to elicit STAT3 phosphorylation whether exposed to OPC or astrocyte-conditioned medium (unpublished data). Altogether, our studies demonstrate that CNTF does not elicit STAT3 phosphorylation in either rat or murine microglia, much to our surprise.

In summary, although CNTF is known for its trophic effects on neurons and oligodendrocytes, it also regulates neuroinflammation. Our results shed light on how CNTF in combination with its soluble receptor serves as a pro-inflammatory signal to enhance central and peripheral immune responses. In particular, our data show that CNTF serves as a weak pro-inflammatory signal to enhance the production of Cox-2, PGE_2 _and CD40 in microglia. CNTF was evaluated in clinical trials, which were halted due to unexpected side effects. Our results provide new data and new insights into possible complications of utilizing CNTF as a therapeutic treatment for motor neuron diseases. In particular, in addition to the weight loss that has been documented [61-63]. CNTF treatment could raise central and peripheral immune responses and lead to a more inflamed environment, which may counteract its trophic activity on neurons and oligodendrocytes.

## Abbreviations

LIF: leukemia inhibitory factor; CNTF: ciliary neurotrophic factor; CNTFRα: CNTF receptor α: IFNγ: interferon-γ; MAPK: mitogen-activated protein kinase; JAK: Janus kinases; STAT: signal transducers and activators of transcription; APC: antigen presenting cell; MHC: major histocompatibility complex; MS: multiple sclerosis; EAE: experimental animal encephalomyelitis; AD: Alzheimer's disease; GMCSF: granulocyte-macrophage colony stimulating factor; gp130: glycoprotein 130; Cox-2: cyclooxygenase-2.

## Competing interests

The authors declare that they have no competing interests.

## Authors' contributions

H-WL participated in the design of the study, prepared the primary cultures, performed western blot and flow cytometry, PGE2 ELISA analyses, and prepared the manuscript. MRJ carried out the 2D electrophoresis and mass spectrometric analysis. SWL and HL supervised the studies. All authors have read and approved of the final manuscript.
